# Investigating the Role of Nandrolone Decanoate in the Management of Osteosarcopenia in Postmenopausal Women: A Prospective Observational Study

**DOI:** 10.7759/cureus.84726

**Published:** 2025-05-24

**Authors:** Bharat Dave, Sandesh Agarawal, Ajay Krishnan, Shivanand Mayi, Ravi R Rai, Mirant B Dave, Abhijith Anil

**Affiliations:** 1 Spine Surgery, Stavya Spine Hospital and Research Institute, Ahmedabad, IND

**Keywords:** lean body mass, nandrolone, osteoporosis, osteosarcopenia, sarcopenia

## Abstract

Introduction

Sarcopenia and osteoporosis are progressive, age-related conditions that often coexist in older adults, resulting in a combined syndrome known as osteosarcopenia. This condition is characterized by a decline in bone mineral density (BMD) and skeletal muscle mass, contributing to increased functional impairment and diminished quality of life. While pharmacological agents such as alendronate are used in the management of osteoporosis, there are currently no Food and Drug Administration (FDA)-approved treatments that specifically address sarcopenia. This study explores the therapeutic potential and safety of incorporating nandrolone decanoate, an anabolic steroid, into an alendronate regimen for the management of osteosarcopenia.

Methodology

This prospective, single-center study was conducted at a tertiary care spine hospital in India. The study enrolled 100 postmenopausal women between the ages of 45 and 80, all diagnosed with osteoporosis using dual-energy X-ray absorptiometry (DEXA). Each participant received intramuscular nandrolone decanoate at a dose of 50 mg every three weeks for 12 weeks, followed by four-weekly injections. This regimen was administered alongside weekly oral alendronate for one year. All participants also received routine calcium and vitamin D supplementation. Assessments were performed at baseline and after 12 months, including DEXA scans to evaluate BMD, lean body mass, and fat mass. Patient-reported outcomes were captured using the Oswestry Disability Index (ODI), Visual Analog Scale (VAS), and a standardized Quality of Life Score (QoLS). Adverse events were recorded at 3, 6, and 12 months to evaluate treatment safety.

Results

A total of 89 out of the 100 enrolled participants completed the study. Baseline assessments revealed median BMD T-scores of -3.1 at the lumbar spine and -2.9 at Ward's triangle. The mean lean body mass was 31,984.07 g, while the mean total body fat mass was 25,966.96 g. After 12 months, BMD significantly improved at both the lumbar spine and hip. Lean body mass increased from 31,984 to 33,062 g, while total fat mass decreased slightly, although the change was not statistically significant. Patient-reported outcomes revealed substantial improvement in back pain (VAS), disability (ODI), and QoLS. No adverse events were recorded throughout the study period.

Conclusion

The incorporation of nandrolone decanoate with alendronate therapy significantly improved lean body mass, BMD, and patient-reported outcomes among postmenopausal women with osteosarcopenia. This therapeutic combination exhibited an excellent safety profile, with no adverse events recorded throughout the study period. These findings highlight the potential of nandrolone decanoate as an effective adjunct in the integrated management of osteoporosis and sarcopenia, contributing to improved overall patient health outcomes. However, further longitudinal studies are required to assess the long-term safety and efficacy of this therapeutic approach.

## Introduction

Sarcopenia is a multifactorial, progressive skeletal muscle disorder characterized by the accelerated loss of muscle mass and functional capacity. It significantly contributes to the onset of frailty, physical disability, and diminished quality of life, particularly among the ageing population. Initially described by Rosenberg in 1988, the term sarcopenia encapsulates the gradual decline in lean body mass, muscle strength, and overall physical performance associated with ageing [1,2]. Key features of sarcopenia include myosteatosis, a pathological condition marked by ectopic fat infiltration within and between muscle fibers, as well as pronounced muscle atrophy. Osteoporosis, similarly prevalent in older adults, is defined by a significant reduction in bone mineral density (BMD) and the deterioration of bone microarchitecture, predisposing individuals to an increased risk of fragility fractures [3]. The World Health Organization (WHO) defines osteoporosis as a condition in which bone mineral density is more than 2.5 standard deviations below the mean for a young, healthy adult [4]. Osteopenia, in contrast, is characterized by a BMD between 1 and 2.5 standard deviations below the mean. At times, osteoporosis and sarcopenia may manifest together in older individuals.

The term osteosarcopenia refers to the coexistence of osteoporosis and sarcopenia. It is defined by the simultaneous presence of low BMD and reduced muscle mass, often accompanied by functional impairment. Epidemiologically, osteosarcopenia is an increasing concern, particularly in older adults. Numerous studies have explored the relationship between sarcopenia and osteoporosis, with the prevalence of osteosarcopenia ranging from 5% to 37% in the general population. Among individuals with low-energy osteoporotic fractures, the prevalence of sarcopenia ranges from 7.8% to 58% in women and 1.3% to 96% in men [5]. A meta-analysis has indicated a 46% prevalence of sarcopenia in individuals with fractures. As the global population ages, addressing osteosarcopenia has become critical due to its significant impact on physical function and overall quality of life [6]. The prevalence of sarcopenia increases with age, affecting approximately 5%-13% of individuals aged 60 and older, with figures reaching as high as 50% in those aged 80 years and above [7]. The condition is notably more prevalent in older women, primarily due to hormonal changes following menopause [8]. Osteosarcopenia is particularly common in postmenopausal women and older adults with fragility fractures. Studies have shown that osteosarcopenia negatively affects physical function, increasing the risk of falls, disability, and mortality [9]. Additionally, osteosarcopenia contributes to frailty, which exacerbates the challenges faced by older adults, heightening their susceptibility to complications and reducing their quality of life.

While pharmacotherapy for osteoporosis is well-established, no drugs have been specifically approved by the Food and Drug Administration (FDA) to treat sarcopenia. This is largely due to the recent recognition of sarcopenia as a clinical entity. Current treatments for osteoporosis, such as bisphosphonates and denosumab, primarily focus on improving bone mineral density but have little to no effect on muscle mass and function. However, emerging evidence suggests that certain osteoporosis drugs, including denosumab and nandrolone decanoate [8], may provide dual benefits for both bone and muscle health, offering a promising therapeutic strategy for managing osteosarcopenia.

Nandrolone decanoate is a long-acting anabolic androgenic steroid hormone administered via intramuscular injection. It has shown potential in enhancing calcium uptake from the gastrointestinal tract, promoting osteoblast activity, and inhibiting osteoclast activity [10]. Additionally, animal studies have demonstrated that nandrolone decanoate increases the expression of insulin-like growth factor (IGF-1) and osteocalcin, both of which support osteoblast function. It has shown a positive effect on lean body mass, alongside an increase in bone mass, and also helps reduce body fat mass. This study aims to assess the efficacy and safety profile of combining nandrolone decanoate with alendronate in managing osteosarcopenia, with a particular focus on its effects on lean body mass and overall patient outcomes.

## Materials and methods

This was an observational, prospective cohort study conducted at a tertiary care spine hospital in India from September 2019 to August 2020. Ethical approval for the study was granted by the institutional ethics committee (protocol number: SSHRI/CS/NS/DECA penia/BRD/30/07-19). Following written informed consent, 100 postmenopausal female patients, aged between 45 and 80 years, who were diagnosed with osteoporosis based on dual-energy X-ray absorptiometry (DEXA) scanning, were enrolled in the study. The inclusion and exclusion criteria are detailed in Table 1.

**Table 1:** Inclusion and exclusion criteria BMD: bone mineral density, PTH: parathyroid hormone, SERMs: selective estrogen receptor modulators, ULN: upper limit of normal, HIV: human immunodeficiency virus, HCV: hepatitis C virus, HBsAg: hepatitis B surface antigen

Inclusion criteria
Women: 45-80 years of age
Ambulatory patients
Patients with BMD value consistent with a T-score between ≤ -2.5 at either Ward's triangle or lumbar spine or greater trochanter or distal one-third radius
Written informed consent from the patient
Exclusion criteria
History of hypersensitivity to nandrolone or alendronate, or any of its excipients
Patients with any metabolic bone diseases, such as, but not limited to, osteomalacia or osteogenesis imperfecta, Paget's disease, Cushing's disease, or hyperprolactinemia
Patients with any malignancy
Patients with severe, untreated hypercalcemia or hypocalcemia
Uncontrolled hyperthyroidism or hypothyroidism, except for patients on stable thyroid hormone replacement therapy for the last year
History of hyperparathyroidism
History of any surgery within three months
Administration of bone metabolism drugs within the last six weeks, such as anabolic steroids or testosterone, PTH or PTH derivatives, e.g., teriparatide, glucocorticoid (S mg prednisone equivalent per day for more than 10 days), systemic hormone replacement therapy, SERMs, e.g., raloxifene, tibolone, calcitonin, anticonvulsants (except benzodiazepines), chronic systemic ketoconazole, androgens, adrenocorticotropic hormone, cinacalcet, aluminum, lithium, protease inhibitors, methotrexate, gonadotropin-releasing hormone agonists
Received any solid organ or bone marrow transplant or on chronic immunosuppression for any reason
Patients with hepatic dysfunction (serum transaminases: 2: 3 x ULN, alkaline phosphatase or bilirubin: 2: 2 x ULN) or renal dysfunction (serum creatinine ~ 2 mg/dL)
Patients with clinically significant uncontrolled systemic diseases such as cardiovascular, renal, neurological, psychiatric, endocrine, immunological, or hematologic disorders, or malignancy
Known to have tested positive for HIV, HCV, or HBsAg
Participation in another clinical trial in the past three months
History of alcohol or drug abuse

Participants received intramuscular nandrolone decanoate 50 mg every three weeks for an initial period of 12 weeks, followed by a maintenance dose of 50 mg every four weeks for the subsequent 36 weeks. In addition, all participants were prescribed oral alendronate 70 mg once weekly for the entire duration of the study. Supplementation with calcium and vitamin D was provided consistently throughout the study period. Participants were reassessed at the 12-month follow-up. The baseline characteristics of the cohort are summarized in the Results section. At baseline and following the 12-month treatment period, each patient underwent a DEXA scan to assess bone mineral density at the lumbar spine and hip. Measurements of lean body mass and total body fat mass were also recorded from the DEXA scans. Patient-reported outcomes, including the Oswestry Disability Index (ODI), Visual Analog Scale (VAS), and Quality of Life Score (QoLS), were assessed at baseline and the 12-month follow-up. Adverse effects were systematically monitored at the three-month, six-month, and 12-month follow-up visits. Specifically, participants were questioned about the onset of virilizing features, dermatologic changes, and mood disorders. Additionally, they were evaluated for the development of dyslipidemia and hypertension during each follow-up visit.

Descriptive statistics were used to summarize baseline characteristics. Continuous variables were expressed as mean ± standard deviation for normally distributed data or median (interquartile range (IQR)) for non-normally distributed data, while categorical variables were presented as frequencies and percentages. The normality of data was assessed using the Kolmogorov-Smirnov test (with Lilliefors modification). To assess changes in BMD, lean body mass, and total body fat mass from baseline to 12 months, the Wilcoxon signed-rank test was used for non-normally distributed data, while the paired t-test was employed for normally distributed continuous data. The significance of changes in patient-reported outcomes (ODI, VAS, and QoLS) was also evaluated using the paired t-test. A p-value of <0.05 was considered statistically significant for all analyses. Adverse effects were evaluated using descriptive statistics, and the incidence of adverse events across follow-up visits was analyzed using chi-square tests for categorical variables. Statistical analyses were performed using SPSS version 25 (IBM Corp., Armonk, NY). All statistical tests were two-sided.

## Results

**Figure 1:**
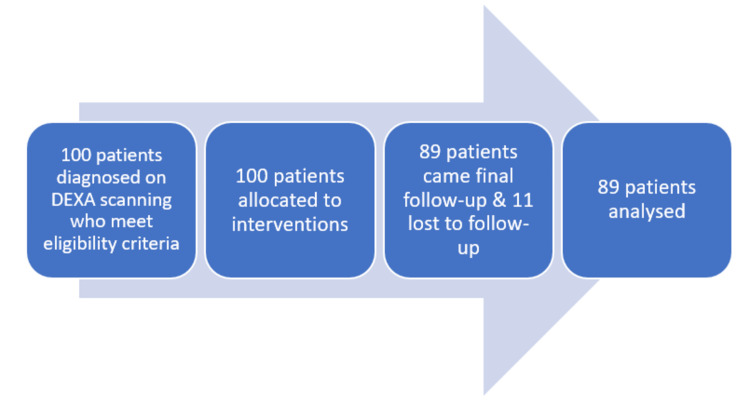
Flow diagram showing the details of the participants in the study DEXA: dual-energy X-ray absorptiometry

**Figure 2:**
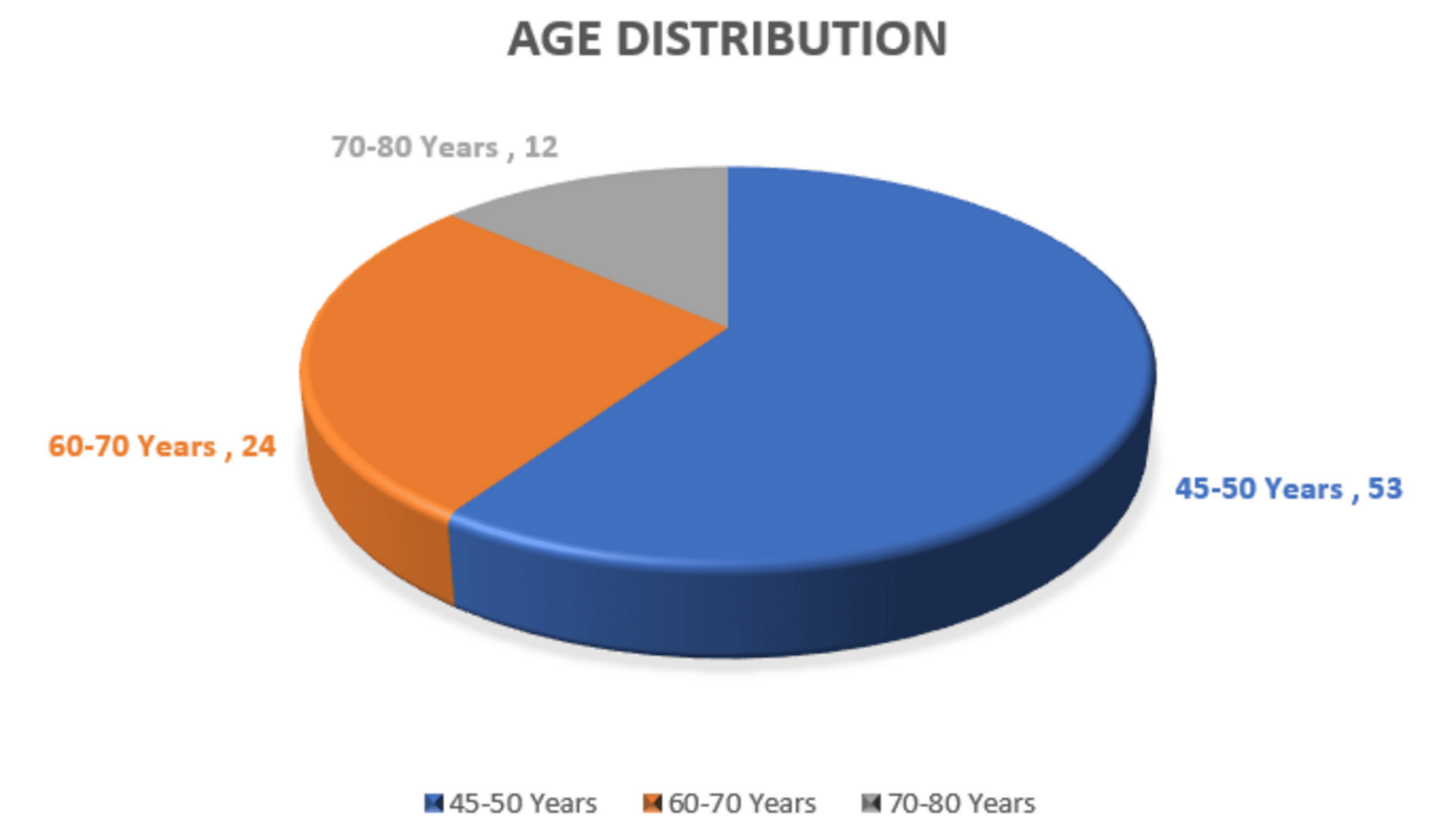
Age distribution

Of the 100 female patients initially enrolled in the study, 11 were lost to follow-up, resulting in 89 participants who completed the study. The details of participant recruitment and retention are illustrated in Figure 1. The mean age of the participants was 54.5 years, with a range of 45-80 years, as illustrated in Figure 2.

At baseline, the 89 patients exhibited a median BMD T-score of -3.1 at the lumbar spine and -2.9 at Ward's triangle. Additionally, the median BMD T-scores at the neck of the femur and greater trochanter were -2.1 and -1.9, respectively. The DEXA scan also revealed a mean lean body mass of 31,984.07 ± 6,710.70 g and a total body fat mass of 25,966.96 ± 9,293.26 g. The cohort had a mean VAS score of 6 ± 1.76 for back pain. Furthermore, the mean ODI was 38.22% ± 11.3%, and the mean QoLS was 37.25 ± 6.93, as illustrated in Table 2, Table 3, and Table 4, respectively. 

**Table 2:** Bone mineral density at baseline visit BMD: bone mineral density, IQR: interquartile range

BMD at baseline visit	Median	IQR
Lumbar spine	-3.10	-2.60-(-3.75)
Ward's triangle	-2.90	-2.3-(-3.6)

**Table 3:** Patient-reported outcome measures at baseline visit ODI: Oswestry Disability Index, VAS: Visual Analog Scale, QoLS: Quality of Life Score

Patient-reported outcome measures at baseline visit	Mean	Standard deviation
ODI score	38.22	11.30
VAS score	6.42	1.76
QoLS	37.25	6.93

**Table 4:** Lean body mass and body fat at baseline visit

Lean body mass and body fat at baseline visits	Mean	Standard deviation
Lean mass	31,984.07 g	6,710.70
Fat mass	25,966.96 g	9,293.26

Upon reassessment after 12 months of therapy, a statistically significant improvement in BMD was observed across all measured sites. The median BMD T-score at Ward's triangle improved from -2.9 to -2.60, at the lumbar spine from -3.1 to -2.9, and at the neck of the femur and greater trochanter from -1.95 to -1.85, as depicted in Table 5. A significant increase in lean body mass was noted, with a mean value rising from 31,984 g to 33,062 g (±7,473.24). The total body fat mass decreased from 25,966.96 g to 25,158.92 ± 9,057.21 g, as depicted in Table 6, although this change was not statistically significant. It is important to note that minimal clinically important differences in BMD T-scores or lean body mass remain undefined in the literature [11].

**Table 5:** Change in bone mineral density with nandrolone decanoate BMD: bone mineral density, IQR: interquartile range

Site	Number	Pre-treatment		Post-treatment		p-value
		BMD		BMD		
		Median	IQR	Median	IQR	
Ward's triangle	89	-2.90	-2.3-(-3.6)	-2.60	-1.90-(-3.25)	<0.001
Lumbar spine	89	-3.10	-2.60-(-3.75)	-2.90	-2.10-(-3.35)	<0.001

**Table 6:** Change in lean body mass and total body fat after therapy with nandrolone decanoate

Variable	Number	Pre-treatment		Post-treatment		p-value
		Mean (grams)	Standard deviation	Mean (grams)	Standard deviation	
Lean body mass	89	31,984.07	6,710.70	33,062.09	7,473.24	0.039
Total body fat	89	25,966.96	9,293.26	25,158.92	9,057.21	0.91

In terms of patient-reported outcomes, the VAS score for back pain showed a significant reduction, decreasing from 6 to 3. The ODI also demonstrated a statistically significant decrease, from 38.22% to 24.51% (± 9.55%), reflecting an average change of 5.99, which is clinically significant [12]. The QoLS improved from 37.3 ± 6.93 to 51.69 ± 9.04, with this improvement being statistically significant and exceeding the minimal clinically important difference of 7-8 points, representing a 60% improvement in symptoms [13]. These changes in patient-reported outcome measures are summarized in Table 7.

**Table 7:** Patient-reported outcome measures before and after therapy with nandrolone decanoate VAS: Visual Analog Scale, ODI: Oswestry Disability Index, QoLS: Quality of Life Score

Patient-reported outcomes	Pre-treatment		Post-treatment		p-value
	Mean	Standard deviation	Mean	Standard deviation	
VAS score	6.42	1.76	3.23	1.9	<0.001
ODI score	38.22	11.3	24.51	9.55	<0.001
QoLS	37.25	6.93	51.69	9.04	<0.001

No patients sustained fragility fractures during the study. Additionally, there were no reports of adverse events during the study period. Remarkably, no exacerbation of symptoms was observed in the study population.

## Discussion

Anabolic steroids, specifically nandrolone decanoate, have attracted significant research interest due to their potential to enhance both BMD and lean muscle mass in individuals with osteoporosis. This synthetic anabolic steroid has been the subject of extensive research focusing on its effects on body composition, with a growing body of evidence highlighting its ability to increase lean body mass and improve BMD. In a randomized, double-blind, placebo-controlled study, Frisoli et al. (2015) investigated the effects of nandrolone decanoate on body composition in postmenopausal women with osteoporosis. Their results demonstrated a significant increase in lean body mass at both the one- and two-year follow-ups, with mean changes of 6.2% (± 5.8%) and 11.9% (± 29.2%), respectively, in the treatment group [14]. Additionally, nandrolone decanoate led to substantial improvements in BMD at both the lumbar spine and hip. A secondary outcome of the study was a significant increase in hemoglobin levels, suggesting a broader systemic effect of the treatment. Similarly, Hassager et al. (2012) assessed the impact of nandrolone decanoate on body composition and plasma lipid metabolism in postmenopausal women with osteoporosis. Their findings revealed an average increase of 4 kg in lean body mass following 12 months of treatment with 50 mg nandrolone decanoate administered every three weeks. Furthermore, a reduction in fat mass was observed, further supporting the anabolic effects of nandrolone in this patient population [15]. These results are consistent with those observed in our study, where patients demonstrated an average increase of 1.2 kg in lean body mass over a 12-month period. However, to the best of our knowledge, no studies have yet analyzed the combination therapy of alendronate and nandrolone decanoate.

The anabolic effects of nandrolone decanoate are not confined to individuals with osteoporosis. Johansen et al. (2011) examined the impact of nandrolone decanoate on muscle wasting in patients on dialysis, reporting a significant increase of 4.5 kg in lean body mass after six months of weekly nandrolone injections [16]. These findings suggest that the anabolic properties of nandrolone decanoate extend beyond osteoporosis and may be beneficial for addressing muscle wasting in various clinical conditions. Furthermore, nandrolone decanoate has demonstrated positive effects on bone health, as evidenced by a study conducted by Flicker et al. (2009), who administered 20 doses of nandrolone decanoate over two years to postmenopausal women with osteoporosis. Significant increases in both bone mineral content and BMD at the lumbar spine and hip were observed, as assessed by DEXA [17]. Our study supports these findings, showing improvements in BMD at multiple skeletal sites following nandrolone decanoate treatment.

A key aspect of our investigation was the concurrent use of nandrolone decanoate with alendronate therapy. Alendronate, a bisphosphonate widely prescribed for osteoporosis, is effective in increasing BMD but has a limited impact on sarcopenia, a condition commonly associated with osteoporosis. The prevalence of sarcopenia in patients with osteoporosis ranges from 5% to 37%, and it is increasingly recognized as a significant contributor to falls and fractures in this population [18]. Nandrolone decanoate represents a promising therapeutic approach to address both bone fragility and sarcopenia, potentially improving outcomes by targeting these two critical issues. Binkley et al. (2009) introduced the concept of osteosarcopenia, recognizing the dual burden of osteoporosis and sarcopenia in older adults and its detrimental effects on health outcomes, including falls and fractures [19]. Given the coexistence of these conditions, the combination of nandrolone decanoate with alendronate exerts additional benefits in treating both bone and muscle loss, offering a more comprehensive approach to managing patients with osteoporosis.

The concurrent use of alendronate in our study introduces complexity in interpreting the observed improvements in BMD, as alendronate alone has well-established benefits for bone mineralization. Thus, future studies should directly compare the effects of nandrolone decanoate combined with alendronate to those of alendronate monotherapy in order to more precisely delineate the individual contributions of each treatment. Furthermore, additional research is needed to compare nandrolone decanoate with other anabolic agents, such as teriparatide and abaloparatide, which have demonstrated efficacy in enhancing BMD and reducing fracture risk [20,21]. Comparative trials involving these agents and nandrolone decanoate will be crucial for identifying the most effective treatment regimen for patients with osteoporosis and concomitant sarcopenia.

The role of nandrolone decanoate in promoting muscle regeneration, an essential component of sarcopenia management, has been highlighted in several studies. Lovato and Lewiecki (2017) investigated the potential of anabolic agents to reverse age-related muscle wasting, suggesting that nandrolone decanoate may be a promising therapeutic candidate for improving sarcopenic outcomes in older individuals [23]. Additionally, Falqueto et al. (2022) found that anabolic steroids positively influenced muscle strength in patients with muscle-wasting conditions, providing further support for their therapeutic application in sarcopenic osteoporosis [24].

Anabolic steroid use is associated with a range of side effects, notably endocrine disorders (virilization, gynecomastia, and hormonal imbalances), cardiovascular issues (e.g., vascular damage and hypertension), dermatological problems (e.g., acne), psychiatric symptoms (e.g., aggression and mood swings), musculoskeletal injuries (e.g., tendon ruptures), and liver damage [25,26]. In a study by Patanè et al., endocrine effects were most common, reported in 61% of treatment cases and 19% of abuse cases. In men, 45% of adverse effects were linked to medical use, while 55% were from illicit use [26]. Remarkably, none of our patients reported any such adverse events.

A significant limitation of our study was the relatively short follow-up period. Long-term studies are required to fully assess the safety profile of extended nandrolone decanoate therapy, particularly concerning cardiovascular risks and potential adverse effects associated with chronic anabolic steroid use [27-29]. Furthermore, clinical guidelines regarding the optimal duration of nandrolone therapy in patients with osteoporosis are currently lacking, and additional trials are necessary to define the minimum effective dose and treatment duration. Another limitation of our study was the absence of a placebo or control group, which would have enabled a clearer comparison of the relative efficacy of nandrolone decanoate versus other treatment modalities. Future research should include larger, multicenter trials with extended follow-up periods to better evaluate the long-term efficacy and safety of nandrolone decanoate, particularly when combined with other established osteoporosis treatments.

## Conclusions

In this study, we identified a notable increase in both BMD and lean body mass in the older adult population following the administration of nandrolone decanoate in combination with alendronate. The rise in lean body mass is primarily attributed to nandrolone decanoate, underscoring its potential as a dual therapeutic agent for addressing both bone fragility and sarcopenia. The observed enhancements in lean body mass and BMD, along with their systemic influence on hemoglobin levels, reinforce the broader therapeutic potential of nandrolone decanoate in treating osteoporotic conditions. The combination of nandrolone decanoate and alendronate offers a promising approach for concurrently targeting both skeletal and muscular degeneration. However, further studies are needed to clarify the specific contributions of each treatment and their mechanism. However, further investigation is warranted to delineate the individual contributions and mechanisms of action of each therapy. Future studies should prioritize comparative efficacy trials evaluating nandrolone decanoate in conjunction with other anabolic agents, such as teriparatide and abaloparatide, with the goal of optimizing treatment strategies for patients with concurrent osteoporosis and sarcopenia. Additionally, long-term, multicenter randomized controlled trials are essential to evaluate the safety profile and sustained efficacy of nandrolone decanoate, particularly concerning potential cardiovascular risks and other adverse effects. As the global prevalence of osteosarcopenia increases, the need for integrated therapeutic strategies becomes more pressing, and nandrolone decanoate may represent an invaluable tool in the comprehensive management of this complex condition.
